# Identification and Fine Mapping of the Recessive Gene *BK-5*, Which Affects Cell Wall Biosynthesis and Plant Brittleness in Maize

**DOI:** 10.3390/ijms23020814

**Published:** 2022-01-12

**Authors:** Qigui Li, Shujun Nie, Gaoke Li, Jiyuan Du, Ruchang Ren, Xiu Yang, Boyan Liu, Xiaolong Gao, Tianjian Liu, Zhiming Zhang, Xiangyu Zhao, Xinzheng Li, Yongxin Nie, Baichen Wang, Haijian Lin, Haiping Ding, Guangtang Pan

**Affiliations:** 1 Maize Research Institute, Sichuan Agricultural University, Chengdu 611130, China; 18408211893@163.com (Q.L.); sjn0923@163.com (S.N.); linhj521@163.com (H.L.); 2 Guangdong Academy of Agricultural Sciences, Crops Research Institute, Guangzhou 510640, China; ligaoke790326@163.com; 3 State Key Laboratory of Crop Biology, College of Life Sciences, Shandong Agricultural University, Taian 271018, China; wsdjy5555555555555@163.com (J.D.); sgsiban2010@163.com (R.R.); byan_liu@163.com (B.L.); 15615748748@163.com (X.G.); ltj3987@163.com (T.L.); zhzhang@sdau.edu.cn (Z.Z.); zhxy@sdau.edu.cn (X.Z.); lxz@sdau.edu.cn (X.L.); jhxnyx@sdau.edu.cn (Y.N.); 4 Institute of Botany, The Chinese Academy of Sciences, Beijing 100081, China; yangxiu@ibcas.ac.cn (X.Y.); wangbc@ibcas.ac.cn (B.W.)

**Keywords:** maize, brittle stalk, cell wall, cellulose synthase catalytic subunit (CESA)

## Abstract

The cellulose of the plant cell wall indirectly affects the cell shape and straw stiffness of the plant. Here, the novel brittleness mutant *brittle stalk-5* (*bk-5*) of the maize inbred line RP125 was characterized. We found that the mutant displayed brittleness of the stalk and even the whole plant, and that the brittleness phenotype existed during the whole growth period from germination to senescence. The compressive strength was reduced, the cell wall was thinner, and the cellulose content was decreased compared to that of the wild type. Genetic analysis and map-based cloning indicated that *bk-5* was controlled by a single recessive nuclear gene and that it was located in a 90.2-Kb region on chromosome 3 that covers three open reading frames (ORFs). Sequence analysis revealed a single non-synonymous missense mutation, T-to-A, in the last exon of *Zm00001d043477* (B73: version 4, named *BK-5*) that caused the 951th amino acid to go from leucine to histidine. *BK-5* encodes a cellulose synthase catalytic subunit (CesA), which is involved with cellulose synthesis. We found that *BK-5* was constitutively expressed in all tissues of the germinating stage and silking stage, and highly expressed in the leaf, auricula, and root of the silking stage and the 2-cm root and bud of the germinating stage. We found that *BK-5* mainly localized to the Golgi apparatus, suggesting that the protein might move to the plasma membrane with the aid of Golgi in maize. According to RNA-seq data, *bk-5* had more downregulated genes than upregulated genes, and many of the downregulated genes were enzymes and transcription factors related to cellulose, hemicellulose, and lignin biosynthesis of the secondary cell wall. The other differentially expressed genes were related to metabolic and cellular processes, and were significantly enriched in hormone signal transduction, starch and sucrose metabolism, and the plant–pathogen interaction pathway. Taken together, we propose that the mutation of gene *BK-5* causes the brittle stalk phenotype and provides important insights into the regulatory mechanism of cellulose biosynthesis and cell wall development in maize.

## 1. Introduction

Plant cell walls are renewable and play an important role in maintaining cell shape and straw stiffness. More importantly, as the skeleton of plants, cell walls greatly affect the lodging resistance of plants, which affects the crop yield [1]. The plant cell wall is mainly composed of three parts: the middle lamella, the primary wall, and the secondary wall. The main common components of the cell wall include cellulose, hemicellulose, pectin, and lignin, with cellulose being the main constituent of the cell wall in most plant species [2,3]. Cellulose is synthesized by the cellulose synthase complex (CSC) on the plasma membrane (PM). The CSC complex exhibits a six-fold symmetry and is known as a “rosette.” Each CSC contains 18–24 cellulose synthase catalytic subunits (CESA) [4]. All discovered CESA proteins are 986–1088 amino acids in length and have the same overall structure, including two N-terminal transmembrane domains (TMDs), six C-terminal TMDs, N-terminal zinc finger domains, and a cytoplasmic catalytic domain between TMD2 and TMD3 [5]. Synthase complexes composed of different CESA subtypes are responsible separately for the cellulose of primary and secondary cell walls.

In fact, in *Arabidopsis*, the synthesis of cellulose in the primary cell wall is carried out by the complex formed by AtCESA1, AtCESA3, and AtCESA6 [6,7], discovered through a variety of mutants [8,9,10,11,12], and by AtCESA2, AtCESA5, and AtCESA9 [13,14], which are classified as AtCESA6-related CESA isoforms due to their similar expression pattern [7]. The cellulose of the secondary wall in *Arabidopsis* is deposited by a complex composed of AtCESA4, AtCESA7, and AtCESA8 [15,16,17,18], whereas in rice, the complex is composed of OsCESA4, OsCESA7, and OsCESA9 [19]. In rice, the mutations of these *CesA* genes produce the *brittle culm* phenotype [20,21,22,23,24,25,26,27,28,29,30,31,32,33], such as mutants *s1-60* [34], *fc16* [35], *fc17* [36], and *tos17* [19]. In total, ten *CesA* genes have been found in *Arabidopsis thaliana* [37,38,39], thirteen in maize, and eleven in rice [40,41], with more genes discovered in barley [42], poplar [43], cotton [44], and many other species.

In maize, there are few studies on the identification and functional analysis of *CesA* genes. Instead, the focus has been on the sequence and expression pattern of the *CesA* gene family [45]. Researchers have isolated 12 *CesA* genes from maize and have divided them into groups according to expression and sequence. ZmCESA1, 7, and 8 form one group, whereas ZmCESA3 and 5 form a second group. ZmCESA10, 11, and 12 may interact with each other to form a functional enzyme complex [46]. Additionally, ZmCESA2 and 6 exhibit their own independent gene expression pattern. In addition to *CesA* genes, maize also has *CesA-like* (*Csl*) genes that are proposed to synthesize other essential non-cellulosic polysaccharides that comprise plant cell walls [47,48,49]. At present, 56 members of the *Csl* gene family have been identified in the maize genome and classified into nine subfamilies: *CslA*, *CslB*, *CslC*, *CslD*, *CslE*, *CslF*, *CslG*, *CslH*, and *CslJ* [49]. Most of them have a relatively high expression level in root and tassel tissues, as revealed by transcriptome data.

Functionally, mazie *CesA* genes also have many brittleness mutants. *brittle stalk-2* (*bk2-ref*) mutant has a mutation in *BK2*, which encodes a Cobra-like protein that is homologous to the rice BC1. It affects stalk strength in maize by interfering with the deposition of cellulose in the secondary cell wall in fiber cells and dramatically reduces tissue mechanical strength [46,50]. In addition, *brittle stalk-4* (*bk4*) is also related to stalk strength and is characterized by highly brittle aerial parts [51]. Because of their influence on the content of cellulose and sugars in the cell wall, these brittleness genes are of great research value for understanding the mechanism of cellulose formation in the plant cell wall.

In this study, we characterized the *brittle stalk-5* (*bk-5*) mutant of the maize inbred line RP125, obtained from an ethylene methyl sulfonate (EMS) mutant library. We found that this mutant displayed brittleness of the stalk and the whole plant, was easy to break, had a thinner cell wall, and had decreased cellulose content compared to the wild type. Genetic analysis indicated that *bk-5* was controlled by a single recessive nuclear gene. Using map-based cloning, we identified the mutation site of *bk-5* that caused the various downstream morphological and physiological changes. The *BK-5* gene encodes a cellulose synthase catalytic subunit (CESA) protein. It was constitutively expressed in all the tissues of the germinating stage and silking stage, and it was mainly localized in the Golgi apparatus, suggesting the protein might move to plasma membrane with the aid of Golgi in maize.

## 2. Results

### 2.1. Phenotypic Characterization of bk-5

**Figure 1:**
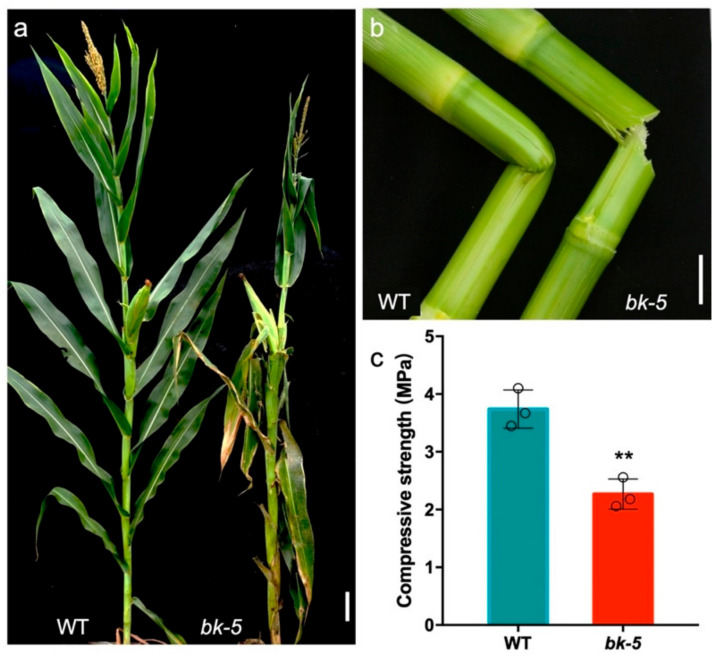
Characterization of *bk-5*. (**a**) Comparison of the appearances of *bk-5* and WT (wild-type: RP125) plants of mature stage. Scale bar: 10 cm. (**b**) Comparison of corn stalk breaking of WT (left) and *bk-5* (right) stems in the jointing stage, an easily broken stalk of *bk-5* was observed. They were broken off with the same force. Scale bar: 5 cm. (**c**) Compressive strength analysis of WT and *bk-5* stems in mature stage, the od circles represent the measured value of each group. The data were presented as values are given as means ± SD and statistically calculated by Student’s *t*-test. ** (*p* < 0.01) indicate significant differences between RP125 and *bk-5*. Compressive strength was calculated based on three plants.

Under natural conditions, we observed that the whole plant of *brittle stalk-5* (*bk-5*) mutant was more brittle than that of the wild type throughout the growth period. In addition, the leaves of the *bk-5* mutant were destroyed or broken off the mature stage under natural conditions (Figure 1a). This phenotype also existed in the early seedling stage (Supplementary Figure S1a). In stem-breaking experiments, in which the same part of the jointing stage stem was bent with the same force, the *bk-5* stem was completely broken with a fracture that was neat and smooth, whereas the wild-type stem stayed connected without fracture (Figure 1b and Figure S1b,c). We further found that the *bk-5* plants were also badly destroyed by the strong wind in the natural field (Supplementary Figure S2). Therefore, we measured the compressive strength of the stem and found that the stem of the mutant *bk-5* was 39% weaker (*p* < 0.01) than that of the wild type (Figure 1c). For unbroken plants, the plant height of *bk-5* was 19% smaller (*p* < 0.01) than the wild type, while the ear height was unchanged (Figure 1a and Figure S1d), signaling that the shortening was caused by the upper part of the plant.

All these phenotypes would likely affect the yield under field planting conditions. Therefore, we assessed the phenotypes of the ear and kernel and found no significant change in their appearance. However, the ear and kernel were slightly smaller than those of the wild type (Supplementary Figure S3a–c). Investigating further, we found that the ear length of *bk-5* was 14% smaller (*p* < 0.01) and ear thickness was 15% smaller (*p* < 0.01) than those of the wild type (Supplementary Figure S3d), as well as the kernel length and kernel width. The kernel thickness of *bk-5* had no significant change (Supplementary Figure S3e), but the ear weight was 28% smaller (*p* < 0.01) and 100-grain weight was 7% smaller (*p* < 0.05) than those of the wild type (Supplementary Figure S3f). In conclusion, we found that *bk-5*′s brittleness and reduced compressive strength existed during the whole growth period, and affected the plant’s development, ears, and grain yield.

### 2.2. Changes in the Cell Wall Composition

**Figure 2:**
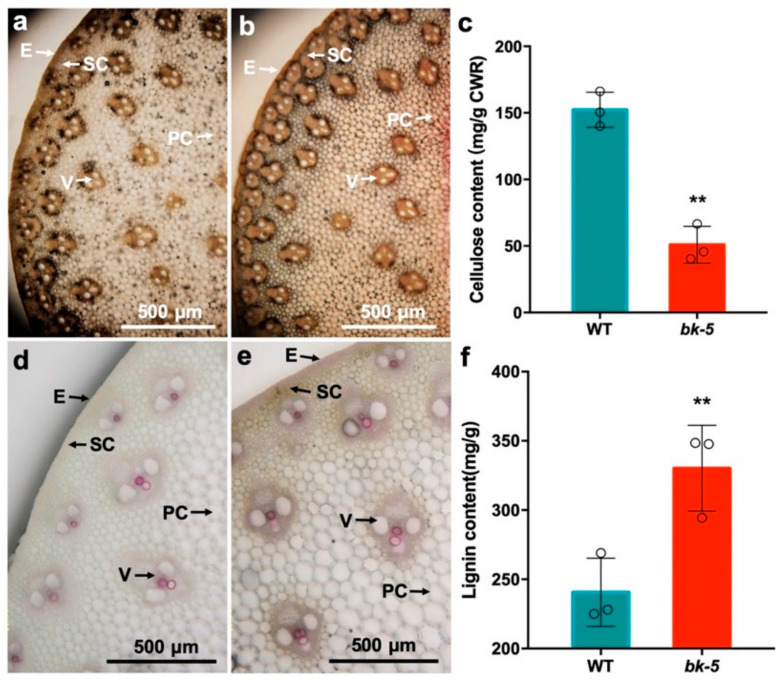
Histochemical staining and measurement of contents. (**a**,**b**) Staining of cellulose and hemicellulose of wild-type (**a**) stalk and *bk-5* (**b**) stalk by zinc chloride iodide solution. The darker the color is, the higher the content of cellulose and hemicellulose is. (**c**) The content of cell wall cellulose of *bk-5* and wild-type stalks. (**d**,**e**) Staining of lignin of wild-type (**d**) stalk and *bk-5* (**e**) stalk by phloroglucinol solution. Pink represents the staining of lignin (the darker pink the color, the higher the content). E, epidermis; PC, parenchyma cells; SC, sclerenchyma cells; V, vascular bundle. (**f**) The content of cell wall lignin of *bk-5* and wild-type stalks. All the samples were from the plants at jointing stage. The stalk uses the third internode above ground. The od circles represent the measured value of each group. The data were presented as values are given as means ± SD and statistically calculated by Student’s *t*-test. ** (*p* < 0.01) indicate significant differences between RP125 and *bk-5*. Three biological repetitions each group were detected.

The reduced compressive strength and cell wall thickness suggested that the cell wall composition in the mutant plant was altered. Cell walls mainly include cellulose, hemicellulose, pectin, and lignin, with cellulose being the main constituent. Therefore, we stained the cell wall of the jointing stage for cellulose, hemicellulose, pectin, and lignin, and found that the total cellulose and hemicellulose content was lower than that of the wild type (Figure 2a,b). To quantify this reduction, we extracted the cell wall cellulose from *bk-5* and wild-type stalks and found that the cellulose content of *bk-5* was 67% lower (*p* < 0.01) than that of the wild type (Figure 2c). On the other hand, the lignin staining in *bk-5* was darker pink than in the wild type (Figure 2d,e), indicating that the content of lignin in *bk-5* was higher. Interestingly, the determination of lignin showed that the lignin content of *bk-5* was 37% higher than that of the wild type, which further confirmed the result of lignin staining (Figure 2f). These results indicate that the low compressive strength of *bk-5* was mainly caused by the decreased cellulose content, suggesting the mutated gene plays an important role in cellulose biosynthesis.

### 2.3. The Cell Wall Defect in bk-5

**Figure 3:**
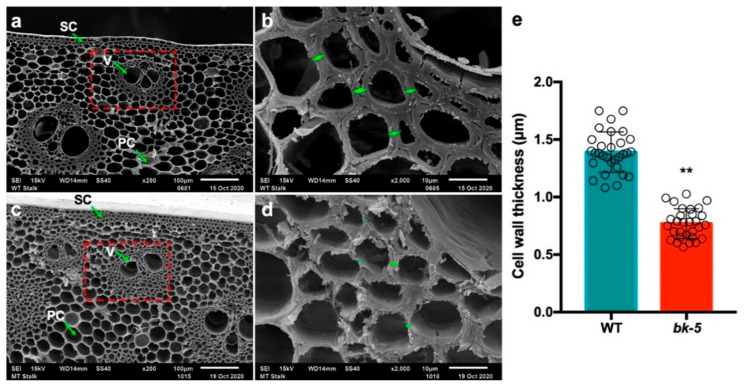
Cross-section of a stalk under a scanning electron microscope. (**a**,**b**) Cross section of a wild-type stalk. (**c**,**d**) Cross section of a *bk-5* stalk. Two different amplifications of ×200 (**a**,**c**) and ×2000 (**b**,**d**) were shown respectively. The third internode above ground of the plant at jointing stage was detected. The green arrow and white text indicate different types of cells, and the red dotted box indicates the indicates the enlarged viewing area. SC, sclerenchyma cells; V, vascular bundles; PC, parenchyma cell. (**e**) Cell wall thickness of *bk-5* and wild-type vascular bundle cells that near the sclerenchyma layers. Thirty values of wild-type and *bk-5* were determined, respectively. The od circles represent the measured value of each group. The data were measured and calculated by Image J. The data was presented as values are given as means ± SD and statistically calculated by Student’s *t*-test. and ** (*p* < 0.01) indicate significant differences between wild-type and *bk-5*.

The reduced compressive strength in *bk-5* might derive from changes in cell wall structure. Thus, we examined the cross section of stalks at the jointing stage using a scanning electron microscope. In the wild-type plant, the cell walls of the vascular bundle, which provide the main structural support for the plant body, are strikingly thick, and the cell wall structure is complete and smooth (Figure 3a,b). By contrast, in *bk*-5, no significant thickening of the vascular bundle’s cell walls was observed, and the cell wall structure was incomplete and rough (Figure 3c,d). Furthermore, we quantified the cell wall thickness of *bk-5* and wild-type vascular bundle cells near the sclerenchyma layers and found that the vascular bundle cell wall thickness of *bk-5* was significantly lower than that of wild type (Figure 3e). On the other hand, no obvious difference in sclerenchyma cells and parenchyma cells were observed (Figure 3a,c) between the wild type and *bk-5*. These results demonstrate that the reduced compressive strength in *bk-5* was due to the defect in the vascular bundle cell wall.

### 2.4. Genetic Analysis and Fine Mapping of bk-5

**Figure 4:**
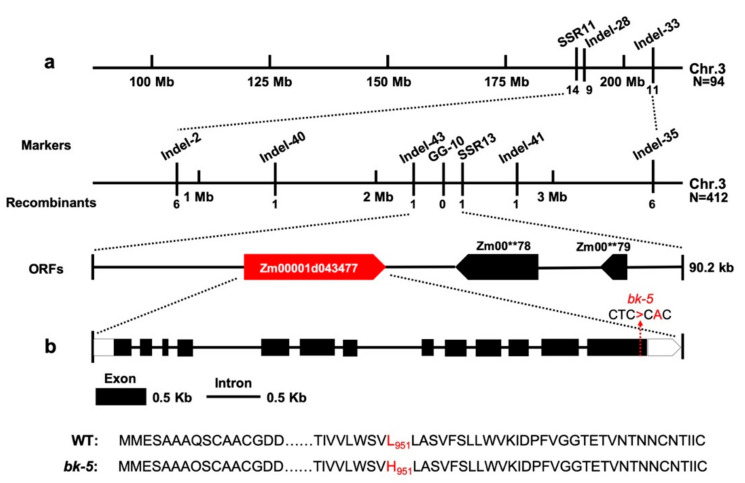
Map-based cloning and identification the gene of *bk-5* using B73 × *bk-5* F2 segregation populations. (**a**) Map-based cloning of *bk-5. bk-5* was preliminary mapped between the flanking markers SSR11 and Indel-33 on chromosome 3 using B73 × *bk-5* F2 segregation populations, with an estimated length of 16.3 Mb (n = 94 individuals with zebra leaf phenotype). *bk-5* was final mapped between molecular markers Indel-33 and SSR13, with an estimated length of 90.2-Kb (n = 412 individuals with brittle stalk phenotype). The target region contained three ORFs based on the genome sequence. (**b**) The gene structure and identification of the mutant gene by sequencing. There was a mutation from T to A on the last exon of gene *Zm00001d043477* (T001). The mutation caused the 951th amino acid mutation of Leu (L) to His (H). ORFs, open reading frames. N, number of F2 plants.

When the *bk-5* mutant was crossed with WT RP125 and the F1 plants were selfed to make the F2 seeds, mutated kernels segregated on F2 ears with a 1:3 ratio, suggesting that the phenotype was controlled by a single recessive mutation (Supplementary Table S1). In a preliminary screening, the DNA of twenty brittle individuals and twenty normal individuals from a segregating F2 population were separately pooled, along with pools of B73 and *bk-135*, and screened with 158 simple sequence repeat (SSR) and insertion/deletion (indel) markers (Supplementary Figure S4). Using these pairs of SSR markers and 94 B73 × *bk-5* F2 individuals, we preliminarily mapped *bk-5* between the flanking markers SSR11 and Indel-33 on chromosome 3 to a region about 16.3 Mb long (Figure 4a, Supplementary Table S2). Based on the published RP125 genome [52], more SSR/indel molecular markers were developed and an additional 412 mutants were also used to fine-map the mutation to a 90.2-Kb region (Figure 4a, Supplementary Table S3). Based on the reference genome, three putative open reading frames (ORFs) were located within this region (Figure 4a). Direct Sanger sequencing of all the ORFs within the mapped region in the *bk-5* mutant and sequence analysis between the *bk-5* and WT RP125 genomes revealed a single non-synonymous missense mutation in the last exon of *Zm00001d043477* (B73: version 4) (Figure 4a,b). The mutation was a T-to-A substitution that changed the 951th amino acid from leucine (L) to histidine (H). Three additional allelic mutants with similar phenotypes and carrying molecular lesions in the same site, with the same mutation (T-to-A), were identified from our EMS mutant collection (Figure 4b and Figure S5), thus confirming that the correct gene was cloned.

### 2.5. Sequence Alignment of the BK-5 Gene

**Figure 5:**
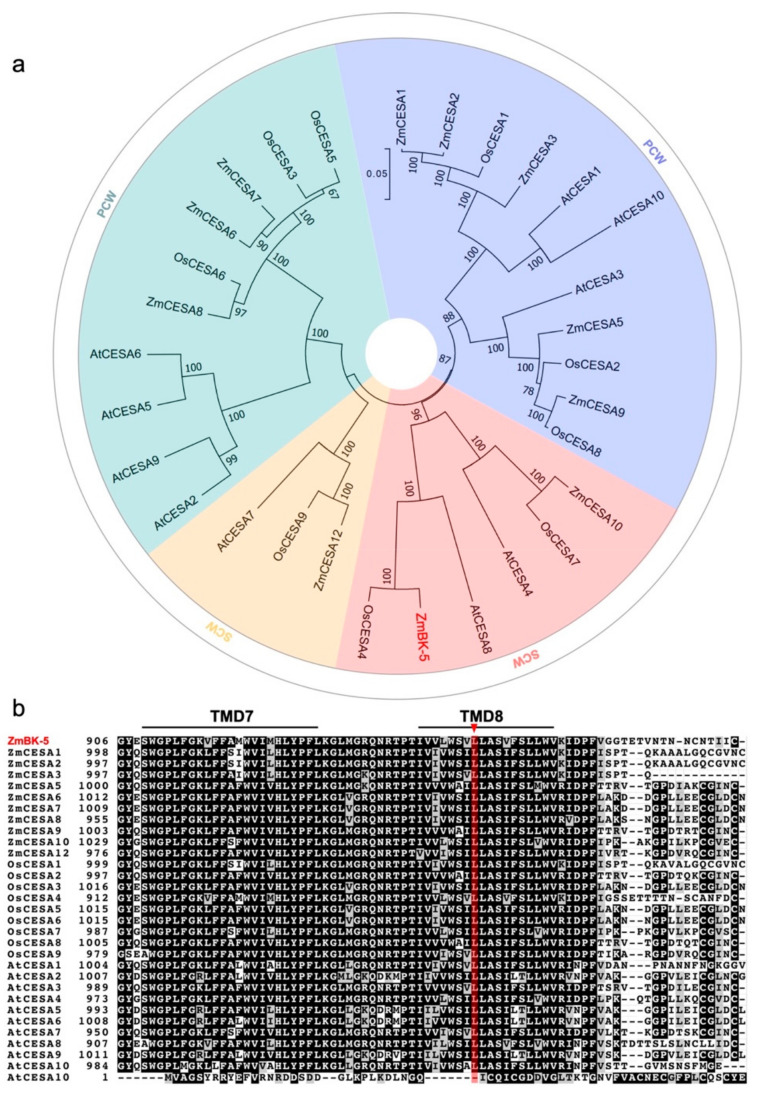
Evolutionary relationships and sequence analysis of BK-5. (**a**) Evolutionary relationships of CESA family. The evolutionary history was inferred using the Neighbor-Joining method. The optimal tree with the sum of branch length = 1.88966750 is shown. The tree is drawn to scale, with branch lengths in the same units as those of the evolutionary distances used to infer the phylogenetic tree. The evolutionary distances, in the units of the number of amino acid substitutions per site, were computed using the JTT matrix-based method. All positions containing gaps and missing data were eliminated. The red diamond represents the branch where ZmBK-5 was located. Four different colored backgrounds represent different primary branches, green and blue backgrounds proteins belong to primary cell wall (PCW), and yellow and pink background proteins belong to secondary cell wall (SCW). The analysis involved 30 amino acid sequences. Evolutionary analyses were conducted in MEGA7. (**b**) Sequence alignment analysis of ZmBK-5 and its homologues. The sequence alignment was completed by ClusterW module of MEGA7, and the similarity value was obtained by ClusterW online (https://www.genome.jp/toolsbin/clustalw, 29 October 2021). The sequence was visualized by boxshade (https://embnet.vi-talit.ch/software/BOX_form.html, 29 October 2021). The red bottom line stands for Cation_ efflux domain, forecast through Pfam (http://pfam.xfam.org/search/sequence, 29 October 2021). The red triangle represented all allelic mutation sites.

The BK-5 protein has 983 amino acids and eight putative transmembrane domains (TMDs): two domains clustered near the hydroxyl terminus and six domains clustered near the carboxyl terminus (Supplementary Figure S6). There is a RING-type zinc finger in the N-terminal region, which might mediate the interaction between BK-5 and other CESA subunits. To identify the homologs of the *BK-5* gene in other plant species, the related protein sequences were downloaded from the NCBI database and compared using phylogenetic analysis and protein alignment. We found that *BK-5* was predicted to encode a cellulose synthase catalytic subunit (CESA) protein belonging to the CESA family and that BK-5 shared a high sequence similarity with its homologs in maize and other species. Significantly, it has a high protein sequence similarity to two CESA proteins, OsCESA4 (92.06%) and AtCESA8 (72.33%), in *Oryza sativa* and *Arabidopsis thaliana* (Figure 5a), both of which are involved in synthesizing the cellulose of the secondary cell wall, i.e., a function of *BK-5*. In addition, multiple sequence alignment revealed that *BK-5*’s sequence is highly conserved among different species. Furthermore, the missense mutation of the *bk-5* mutant is in a highly conserved site in TMD8 of the CESA motif (Figure 5b).

### 2.6. Tissue-Specific Expression Profiles and Subcellular Localization Pattern

**Figure 6:**
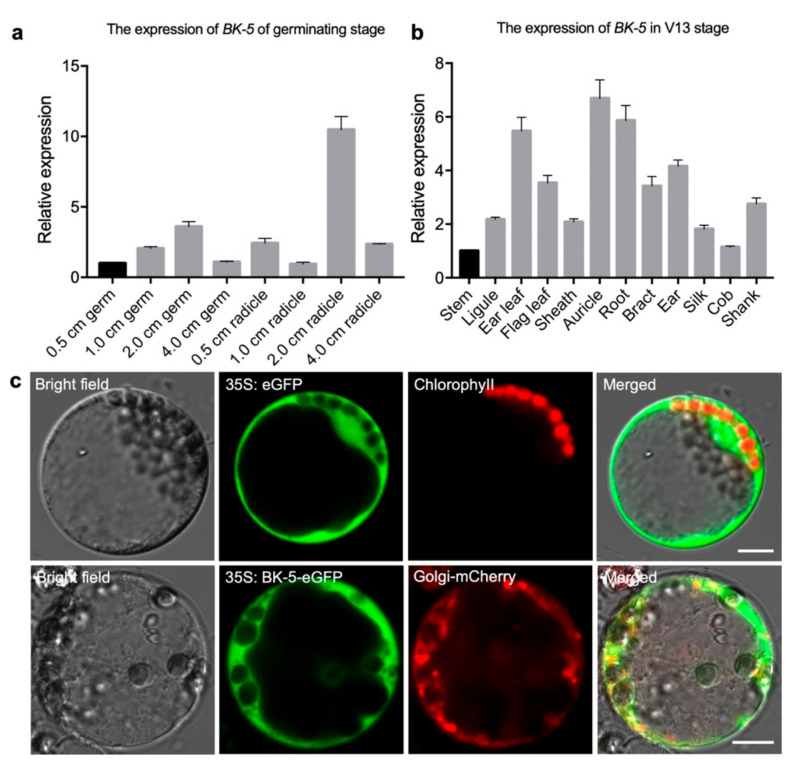
Expression analysis and Subcellular localization of *BK-5*. (**a**) The expression of *BK-5* in 0.5, 1, 2, and 4 cm roots and buds of different germinating stages. *BK-5* high expression in 2-cm roots and buds. (**b**) The expression of *BK-5* in different tissues of V13 stage. Samples of stem, ligule, sheath, and auricle were derived from tenth section. *BK-5* was constitutive expressed in all the tissues. 18S ribosomal RNA (rRNA) was used as an internal control. For each RNA sample, three technical replicates of two tissue samples were performed per stage. Error bars represent standard deviation. (**c**) Subcellular localization of BK-5 in maize protoplasts. From left to right were bright field, green fluorescence, chloroplast/mCherry fluorescence, fusion image. Scale bar: 10 μm.

Because *BK-5* affects plant brittleness when it is mutated, we checked the *BK-5* expression level in different tissues of the whole plant at the germinating stage and silking stage. RT-qPCR revealed that *BK-5* was highly expressed in the 2-cm germ and radicle of the germinating stage (Figure 6a). In the silking stage, *BK-5* was constitutively expressed in all the tissues, including the stem, ligule, ear leaf, flag leaf, sheath, auricle, root, bract, ear, silk, cob, and shank. It was at an especially high expression level in ear leaf, auricle, and root (Figure 6b). To look at its subcellular localization, we fused *BK-5* with enhanced *GFP* (eGFP) to construct a plasmid, *35S: BK-5-eGFP*. This plasmid and a control were transiently expressed in maize protoplasts. Confocal microscopy showed that the green fluorescence of *BK-5-eGFP* was mainly co-localized with the Golgi-mCherry marker and partially localized in cytosol, while the empty eGFP protein was mainly expressed in the cytosol (Figure 6c). These results indicate that the BK-5 protein is mainly located in the Golgi apparatus, suggesting that it may moves to plasma membrane with the aid of Golgi. This result is consistent with other CESA proteins.

### 2.7. RNA-seq Analysis of bk-5

**Figure 7:**
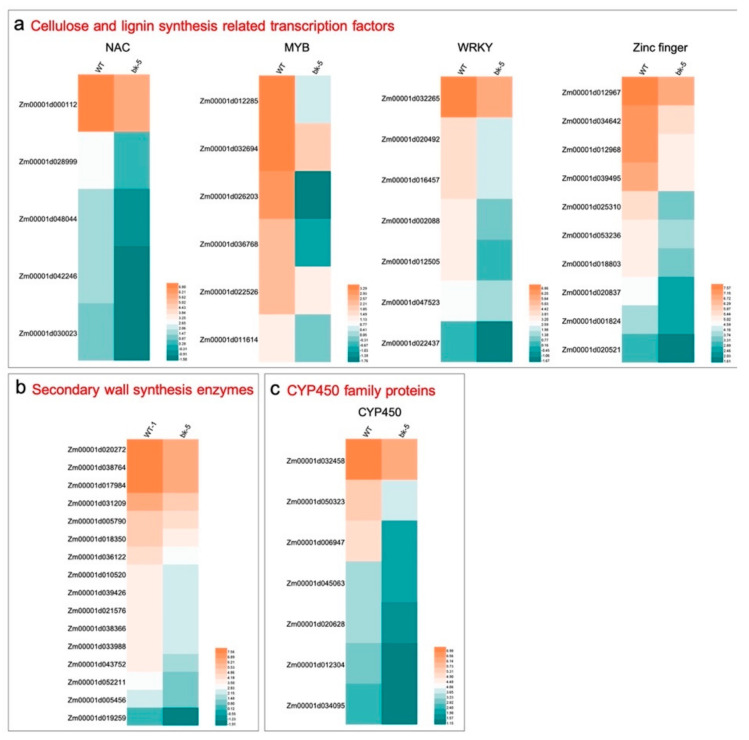
Some differentially expressed gene of *bk-5* and WT. (**a**) Many cellulose and lignin synthesis related transcription factors were down-regulated. This includes NAC (NAM, ATAF1/2, CUC1/2 transcription factor), MYB (v-myb avian myeloblastosis viral oncogene homolog transcription factor), WRKY (transcription factor), and zinc finger. (**b**) Many secondary wall synthesis-related transferase enzymes were down-regulated, most of them were glycosyltransferase. (**c**) CYP450 (cytochrome P450) family proteins were also significantly down-regulated. Each row of the heat map represents the log2 fold values of a differentially expressed gene (green, low expression; orange, high expression). Hierarchical grouping of differentially expressed genes shows clustering analysis by HEML. WT: wild-type.

To identify transcripts that are differentially expressed between *bk-5* and RP125, we employed RNA-seq on the middle part of the eighth leaf in the jointing stage. A total of 26,774 genes were detected, of which 427 were differentially expressed, with 124 genes upregulated and 303 genes downregulated in *bk-5* (Dataset 1). Heatmap more intuitively showed the differential expression of genes between wild-type and *bk-5*, the heatmap hierarchical clustering corroborates that the replicates of the varieties clustered together (Supplementary Figure S7), and there was no significant variability between replicates. According to the evolutionary analysis above, the cellulose synthase catalytic subunits encoded by BK-5 was related to the synthesis of the secondary cell wall (SCW). The main components of SCW are cellulose, hemicellulose, and lignin. In the RNA-seq data, many transcription factors regulating secondary wall synthesis were significantly downregulated in *bk-5* (such as NAC and MYB), the main transcription factors regulating cellulose and lignin synthesis, as well as the WRKY and Zinc finger protein, and transcription factors regulating lignin synthesis (Figure 7a). In addition, many SCW biosynthesis-related transferase enzymes were downregulated in *bk-5*, most of them being glycosyltransferases (GTFs) (Figure 7b). As the main enzymes for the synthesis of cellulose and hemicellulose, GTFs are very important for the biosynthesis of SCW. Interestingly, we found that many CYP450 family proteins involved in basic metabolism and secondary metabolism were also significantly downregulated in *bk-5* (Figure 7c). we randomly selected one gene in each series for quantitative verification, and found that its expression was down regulated in bk-5 in varying degrees, which was consistent with the data of RNA-seq (Supplementary Figure S8). In short, the downregulated enzymes and transcription factors were related to cell wall development and the brittle phenotype of *bk-5*.

We conducted a gene ontology (GO) enrichment analysis (biological process, cellular component, and molecular function) and found that 60–70% of the annotated DEGs (422 genes) belonged to seven categories: the GO biological process categories of “metabolic process” and “cellular process”; the GO cellular component categories of “cell part”, “cell”, and “organelle”; and the GO molecular function categories of “binding” and “catalytic activity” (Supplementary Figure S9a). In terms of metabolic pathways, many DEGs had a role in plant hormone signal transduction (13), followed by starch and sucrose metabolism (7), and plant–pathogen interaction (6) (Supplementary Figure S9b). Thus, the mutation in *BK-5* affected these pathways, which directly or indirectly affect the mutant brittleness phenotype.

## 3. Discussion

Compressive strength is an important agronomic trait in maize production, and it is mainly decided by the cellulose content of plant cell walls. Improving the compressive strength can enhance lodging resistance of maize and increase the crop yield, while reducing the compressive strength causes the brittleness phenotype of the culm or stalk. In practice, it is difficult to find enhancing mutants, but mutants with reduced mechanical strength can be easily found. So far, many brittleness mutants have been identified in *Arabidopsis* and rice, such as the *irregular xylem* (*irx1*, *irx2*, *irx3*, *irx5*) series of mutants in *Arabidopsis* and the *brittle culm* (*bc3*, *bc5*, *bc6*, *bc7*, *bc10*, *bc11*, *bc12*, *bc13*, *bc14*, *bc15*, *bc17*, *bc18*, *bc88*) series of mutants in rice [9,10,11,12,16,17,18,20,21,22,23,24,25,26,27,28,29,30,31,32,33]. Biochemical and molecular characterization of these mutants revealed that the brittleness phenotype was caused by defects in cellulose synthesis, particularly in the *CesA* genes. There are 10, 11, and 13 genes of the *CesA* family in Arabidopsis, rice, and maize, respectively [37,38,39,40,41], and their structures and functions have been widely reported in *Arabidopsis* and rice. For example, AtCESA1, AtCESA3, and AtCESA6 are related to the synthesis of cellulose in the primary wall [6,7], and OsCESA4, OsCESA7, and OsCESA9 participate in the synthesis of the secondary cell wall [19]. However, there are few studies on the identification and function of *CesA* genes in maize just *brittle stalk2* (*bk2*) and *bk4* have been reported [46,50,51]. Other studies mostly focus on sequence analysis and expression pattern research [45]. Maize also has the cellulose synthase-like (ZmCSL) gene family, and studies of that family also focus on annotation and expression in the maize mesocotyl [48,49,53]. Therefore, there are likely many *CesA* genes in maize that remain to be found and studied.

The cellulose of the plant cell wall indirectly affects cell shape and straw stiffness, thus influencing crop yield. The brittle stalk mutants are valuable to the study of cell wall cellulose synthesis and lodging resistance. In this report, we characterized a novel maize brittle stalk mutant, *bk-5*, which is brittle during the whole growth period from germination to senescence. First, we observed the phenotype of stems and leaves of *bk-5* in different developmental stages, such as the germination stage, the early seedling stage, the jointing stage, the silking stage, and the mature stage. The brittle phenotype could be observed as early as in the bud of the germination stage, and the constitutive expression of *BK-5* in different stages of the plants also corresponded to the appearance of the phenotype, showing it has a serious impact on the whole growth period. Second, the phenotypic variation also affected yield-related traits, such as plant height, ear size and weight, grain length, grain width, grain thickness, and 100-grain weight. The result is consistent with previous reports that mutations in *CesA* genes reduce rice height and yield [30,34,36]. Third, we found that the low compressive strength of *bk-5* was mainly caused by the decreased cellulose content, and we also observed that the lignin content showed a significant increase, suggesting that the lignin may also play an important role in plant brittleness. Interestingly, we also observed that cellulose decreased with lignin increased in *brittle culm* series mutants of rice, such as *bc7*, *bc10*, and *bc11* [24,25,26]. Generally, cellulose and lignin are in dynamic equilibrium in plants. A reduction in one will inevitably affect the content of the other, showing a compensatory balance mechanism. However, we pay more attention to the brittleness of the plant, which mainly depends on the content of cellulose, and the content of lignin is more related to the hardness of the plant. In the future, we will pay more attention to the effect of lignin on mechanical strength and brittleness. Lastly, we found the reduction in compressive strength in *bk-5* was due to the defect in the vascular bundle cell wall, with no obvious differences present in the sclerenchyma cells and parenchyma cells of the maize stalk. That is surprising given that mutations in rice CESA proteins cause defects in sclerenchyma and parenchyma cells in the *bc11* and *s1-60* mutant strains [30,34].

CESA is the key enzyme for cellulose synthesis. There are 13 known CESA proteins in maize. This report characterizes a novel CESA protein (BK-5) that is highly conserved among different species and has high sequence similarity with its well-studied homolog, OsCESA4, in rice. They share domains and motifs, such as zinc fingers and eight TMDs, with all plant CESAs [5]. To verify the correlation between gene and phenotype, we identified three allelic mutants from our EMS mutant library. Surprisingly, these had the same mutation site and same mutation as *bk-5*. We speculate that the high mutation rate of this site may be due to the genetic background of RP125. This site is evolutionarily conserved. The substitution of the leucine residue with a histidine residue at the eighth TMD of BK-5 might change the conformational structure of the protein and disrupt its function, leading to an easily identifiable brittle stalk phenotype.

The BK-5 protein mainly localized to the Golgi apparatus but was also present in the cytoplasm, which may be related to the dynamic protein transfer process, in that it is transcribed in the nucleus, processed in the intimal system, and finally transported to the plasma membrane to function. Most cell wall-related proteins are synthesized in the endoplasmic reticulum (ER). Cellulose synthase complexes (CSCs) mature in the Golgi apparatus then translocate to the plasma membrane (PM) to synthesize cellulose. Therefore, most known CESA proteins are located in the plasma membrane and Golgi apparatus. In rice, cellulose synthases (such as *bc11*, OsCESA4) are assembled into CSCs in the Golgi then translocated to the PM. The PM-localized CSCs are responsible for cellulose biosynthesis, while the Golgi-localized glycosyltransferases (such as *bc10*) and hydrolases catalyze the synthesis of noncellulosic polysaccharides and glycoproteins [54,55].

In our RNA-seq data, many enzymes and transcription factors related to cellulose, hemicellulose, and lignin biosynthesis of SCW were significantly downregulated in the *bk-5* mutant. In the GO and KEGG pathway analyses, multiple genes were associated with hormone signal transduction, carbohydrate metabolism, and plant–pathogen interaction. As the first physical barrier against pathogen invasion, the plant cell wall also participates in sensing external pressure and transmitting corresponding signals to stimulate defense responses. Cell wall components are crucial to plant immunity, such that cellulose, hemicellulose, pectin, and lignin have different roles in plant disease resistance [56,57]. Therefore, mutations in genes related to cell wall biosynthesis or modification may change plant disease resistance.

In conclusion, the identification of the *BK-5* gene not only gives a new perspective of genes involved in cellulose biosynthesis, cell wall development, and plant stalk growth in maize, but is also helpful to the development of potential maize varieties through the marker-assisted selection approach using markers developed in this study. Improving the compressive strength will have a significant impact on enhancing lodging resistance and increasing the crop yield.

## 4. Materials and Methods

### 4.1. Plant Materials and Investigation of Agronomic Traits

The *brittle stalk-5* (*bk-5*) mutant, with brittleness of the stalk and whole plant, was isolated from an EMS-induced mutation library of the cultivar RP125 (wild type). The pollen of RP125 was treated with a 0.1% solution of EMS (Sigma-Aldrich, St. Louis, MO, USA, M0880), dissolved in mineral oil (Sigma-Aldrich, St. Louis, MO, USA, M8410) for 30–40 min, then used to pollinate 60–70 ears to induce mutation. With this method, the EMS mutant library of the RP125 background was constructed by our research group [52]. We screened a series of mutants related to *brittle stalk* mutation in the library, and obtained a stable genetic *brittle stalk* mutant, named *bk-5,* through continuous selfing screening. The phenotype of different stages of plants of *bk-5* were observed during the whole growth period in the field, from mature stages to germinating, seedling, jointing, and silking. At the jointing stage, we did the stalk-breaking experiment. At the mature stage, agronomic traits, including compressive strength, plant height, ear height, ear length, ear thickness, kernel length, kernel width, kernel thickness, ear weight, and 100-grain weight, of the mutant and the wild type were measured. Furthermore, *bk-5* was crossed with B73 to construct the F1 and F2 population for genetic analysis and gene mapping.

### 4.2. Determination of Compressive Strength

The whole plants of mature mutant *bk-315* and wild-type RP125 were taken, and 10 whole stems with similar thickness and no diseases and pests were selected, respectively. The stem segments of the third section near the ground were taken and put on the wood bending strength tester (Yuelian, Guangdong, China, YL-1125) for tension test. The parameters were set as follows: gauge distance = 240 mm, and the tension value (n, n) was read on the display. According to the formula: flexural strength = (3*FNmax*d)/(2*(wide/thick2)). This was used to calculate the bending strength of each material.

### 4.3. Histochemical Staining and Measurement of Cell Wall Composition

Histochemical staining was performed to observe the cellulose and hemicellulose of cell walls, and the third internodes from the ground were collected at the jointing stage. The internodes were cut into 60-μm transverse sections by an ultrathin tissue slicer and fixed in cellulose staining solution (zinc chloride iodide) for 5 min (Leagene, Beijing, China, DP0406), then observed under a light microscope (Leica, Wetzlar, Germany). To observe the lignin of the cell walls, phloroglucinol was selected for staining [58]. The third internodes were cut into 60-μm transverse sections, then fixed in 1% phloroglucinol alcohol solution (*v*/*v*) for 2 min (Sigma, St. Louis, MO, USA, 79330). They were washed with 18% HCl for 5 min until the cross section of the sample turned red, and then observed under a light microscope (Leica, Wetzlar, Germany).

In order to measure cellulose and lignin, the third internodes from the ground were collected at the jointing stage. For lignin measurement, we used a lignin measurement kit (Solarbio, Beijing, China, BC4200). Firstly, the internodes were oven-dried at 105 °C for 30 min, oven-dried at 80 °C to a constant weight, milled into powder, and then passed through a 30–50 mesh sieve to determine lignin contents. The perchloric acid and glacial acetic acid were used to extract and determine content. For cellulose content determination, we used the cellulose measurement kit (Solarbio, Beijing, China, BC4280). Cell wall material (CWM) was extracted by weighing about 0.3 g (W1) of the sample, adding 1 mL of extract 1, and homogenizing it quickly at room temperature with a water bath at 90 °C for 20 min, before cooling it to room temperature, centrifuging 6000× *g* at 25 °C for 10 min, and then discarding the supernatant. The precipitate was washed twice with 1.5 mL of extract 1 and acetone twice. The precipitate is the crude cell wall. After 1 mL of extract 2 was added, it and was soaked for 15 h before being centrifuged at 6000× *g* at 25 °C for 10 min. The supernatant was then discarded and the precipitate was dried to obtain cell wall material (CWM). Next, the weight was recorded as W2. Cellulose was extracted by weighing about 5 mg of dried CWM (W3), adding 0.5 mL of distilled water to homogenize thoroughly, transferring the homogenate to the EP tube, distilling water to make the volume to 0.5 mL, placing it in a mixture of ice and water, slowly adding a mix of 0.75 mL, slowing concentrating sulfuric acid, and letting it stand in an ice water bath for 30 min. It was then centrifuged at 8000× *g* for 10 min at 4 °C. Then, the supernatant was taken and diluted with distilled water 20 times, before waiting for the measurement. The color reaction of concentrated sulfuric acid and anthrone was used for absorbance analysis and conversion. The cellulose and lignin content measurements were improved by Updegraff [59] and Ishimaru [60].

### 4.4. Transmission and Scanning Electron Microscopy

The stalks of the third internode above ground of RP125 and *bk-5* at the jointing stage were quickly harvested on ice, and were then immersed in glutaraldehyde (*w*/*v*, 2.5%) solution (Solarbio, Beijing, China, P1126) for fixation. The sample pretreatment was completed through the experimental platform of State Key Laboratory of Shandong Agricultural University. Firstly, the internodes were washed with PBS solution and dehydrated for at least two days in different concentrations of ethanol, including 45% (30 min), 55% (30 min), 70% (12 h), 85% (30 min), 95% (30 min), and 100% (1 h), 100% (6 h), and 100% (12 h). Secondly, they were fixed in propylene epoxide solution (repeated twice; one hour each time). Thirdly, they were put into a mixture of resin and propylene oxide (5 h), and then treated with pure resin overnight. Fourthly, they were treated with new resin for 9 h the next day. Finally, DMP-30 was added to the resin to promote cross-linking. The samples were dried at 36 °C for 10 h, 45 °C for 14 h, and 60 °C for 36 h. For scanning electron microscopy, the fixed thin pieces of internode were critical-point-dried, sputter-coated with gold and observed by scanning electron microscopy (Sigma 500, Oberkochen, Germany, Zeiss) at different amplifications (200×, 500×, 2000×).

For calculating cell wall thickness, 10 cell wall thickness values were measured from 20 adjacent cells around the vascular bundle cavity closest to the thick wall layer of wild type and *bk-5*, in 2000× magnification fields. Each value was divided by 2 to obtain the thickness of the per cell wall. In the same way, we counted 3 fields, obtained 3 × 10 values, and calculated the mean ± SD. The data were measured by Image J (rsb.info.nih.gov/ij, accessed on 29 October 2021).

### 4.5. Genetic Analysis and Gene Mapping of bk-5

For genetic analysis, the segregating F2 population was constructed by crossing the *bk-5* mutant with B73. The genetic analysis was carried out by calculating the segregation ratio of fragile individuals and normal individuals in the F2 generation. A chi-square test was used to test the fit degree (Supplementary Table S1). The DNA of the F2 population from individual plants was used for the final mapping. As a preliminary screen of *bk-5*, twenty fragile individuals and twenty normal individuals from the F2 population were separately mixed into two DNA pools using the BSA strategy [61], followed by B73 and *bk-5*, and the two pools were screened using the simple sequence repeat (SSR) and insertion/deletion (indel) markers to find the preliminary linkage markers (Supplementary Table S2). The preliminary linkage markers were combined with 94 mutant individuals to locate the preliminary mapping interval. Then, by further expanding the population and screening new polymorphic molecular markers, we fine-mapped the linkage genes of mutant phenotype. Molecular markers for fine mapping were designed based on polymorphisms between the RP125 and B73 reference genomes (Supplementary Table S3). To amplify and identify the mutated gene, the gene was amplified from the wild-type and mutant plants (Vazyme, Nanjing, China, P505) and subjected to Sanger sequencing. Primer sequences are provided in Supplementary Table S4.

### 4.6. Protein Sequence Analysis

Protein sequences were aligned using the ClustalW model of MEGA7 [62]. The evolutionary history was inferred using the neighbor-joining method [63]. The tree is drawn to scale, with branch lengths in the same units as those of the evolutionary distances used to infer the phylogenetic tree. The evolutionary distances were computed using the JTT matrix-based method [64] and are in the units of the number of amino acid substitutions per site. All positions containing gaps and missing data were eliminated. The online tool Protter (http://wlab.ethz.ch/protter/start/, 29 October 2021) was used to predict protein transmembrane structure. Protein similarity values were aligned by CLUSTALW (https://www.genome.jp/tools-bin/clustalw, 29 October 2021). The protein sequence similarity data were visualized by BoxShade.

### 4.7. RNA Extraction and RT-qPCR Analysis

Total RNA was extracted from different tissues of the silking stage and from 0.5, 1, 2, and 4 cm roots and buds of the germinating stage of RP125 by the TRI reagent (Sigma, St. Louis, MO, USA, 93289). The cDNA was reverse-transcribed using the PrimeScript RT Reagent Kit with the gDNA Eraser (Perfect Real Time) kit (Takara, Beijing, China, RR047A). Real-time quantitative PCR (RT-qPCR) was performed using the SYBR Premix Ex TaqII (Tli RNase H Plus) kit (Takara, Beijing, China, RR820A) in an Applied Biosystems thermocycler. Primer sequences are provided in Supplementary Table S4.

### 4.8. Subcellular Localization

To determine the subcellular localization of the BK-5 protein, the full-length coding sequence of *BK-5* with no stop codon was amplified then subcloned into the expression vector pM999 with eGFP (enhanced GFP) under the 35S promoter to construct a C-terminal fusion protein *BK-5*-eGFP. Plasmids 35S: *BK-5*-eGFP and 35S: eGFP were transformed into maize protoplasts using the polyethylene glycol (PEG)-mediated transformation method. The fluorescence of eGFP was detected using a laser confocal scanning microscope ZEISSLSM 700 (ZEISS, Jena, Germany). Golgi-mCherry marker for co-localization came from laboratory preservation. The excitation wavelengths of eGFP and chlorophy II were 488 nm, and the receiving wavelengths were 500–530 nm (eGFP) and 600–750 nm (chlorophy II), respectively. The excitation wavelength of mCherry was 580 nm and the receiving wavelength was 590–750 nm.

### 4.9. RNA Sequencing and Data Analysis

For RNA-seq, the middle part of the eighth leaf of mutant *bk-5* and the wild type (RP125) in the jointing stage were sampled and immediately frozen with liquid nitrogen. Three independent replicates were collected for each sample. The total RNA of the leaves was isolated using the TRIzol reagent (Invitrogen, Waltham, MA, USA) according to the manufacturer’s protocol. Three pools of *bk-5* and three pools of RP125 were subjected to standard Illumina library preparation using the NEB Next Ultra RNA Library Prep Kit according to the manufacturer’s protocol. In brief, polyadenylated RNA purification, RNA fragmentation, cDNA synthesis, and polymerase chain reaction (PCR) amplification were all compelted. The cDNA libraries were paired-end sequenced (125 bp) using an Illumina HiSeq2500 (Novogene, Nanjing, China). The reads were filtered against the rDNA using Bowtie2 (https://bowtie-bio.sourceforge.net/bowtie2/index.shtml, 29 October 2021), and the remaining reads were paired-end aligned with STAR against the B73 reference genome of *Zea mays* (AGPv4).

To determine the DEGs, the data were processed using two tests, DEseq2 and EdgeR, using the software packages, Bioconductor and Galaxy. These tests are among the best and most used performance tools for RNA-seq analysis. Fragments per kilobase pair of exon per million fragments mapped (FPKM) was used to normalize gene expression values. A typical cut-off value of FDR < 0.05 was used in the multiple comparison correction process [65,66]. Heatmap clustering analysis was conducted using the ‘Heml’ tool (Heml 1.0.3.7) in a java environment, aiming to find gene expression patterns across the different varieties. For each differentially expressed gene, Gene Ontology (GO) annotation was obtained at a significance level of 5% by the web-based agriGO tool [67]. DEGs were also annotated using KEGG pathway enrichment analysis, which aimed to significantly identify enriched metabolic pathways or signal transduction pathways affected by the *BK-5* mutation. KOBAS 2.0 was used to test the statistical enrichment of DEGs in KEGG pathways [68].

### 4.10. Statistical Analysis

Kernel length, kernel width, and kernel thickness were examined in each individual by randomly selecting 10 kernels from the center of each ear. Compressive strength, ear length, thickness, and weight were calculated based on three biological replicates. Plant height and ear height were calculated based on ten plants. Kernel length, kernel width, and kernel thickness were calculated based on ten kernels. Then, 100-grain weight was calculated based on the 100-kernel weight with three biological replicates. For expression analyses using RT-qPCR, at least three individual plants were pooled as one biological replicate, and four technical replicates were performed for each sample. Statistical calculations were performed in Microsoft Excel. The data were presented as mean ± SD. Statistical analysis of variance was calculated by Student’s *t*-test. The mean values for each measured parameter were compared using two-way analysis of variance or two-tailed, two-sample Student’s *t*-test, as appropriate. ns indicates no significant change, * (*p* < 0.05) and ** (*p* < 0.01) indicate significant differences.

## Data Availability

RNA datasets reported in this study have been deposited in GenBank (NCBI) with the following accession code: PRJNA777009.

## Supplementary Materials

The following supporting information can be downloaded at: https://www.mdpi.com/article/10.3390/ijms23020814/s1.
