# Supplementary material for: Identification and Fine Mapping of the Recessive Gene BK-5, Which Affects Cell Wall Biosynthesis and Plant Brittleness in Maize
Source: Int J Mol Sci. 2022 Jan 12;23(2):814. doi: 10.3390/ijms23020814 (PMC8775815; doi:10.3390/ijms23020814)
Supplement: Supplementary file 1 [file ijms-23-00814-s001.zip › ijms-1525941-supplementary.pdf]

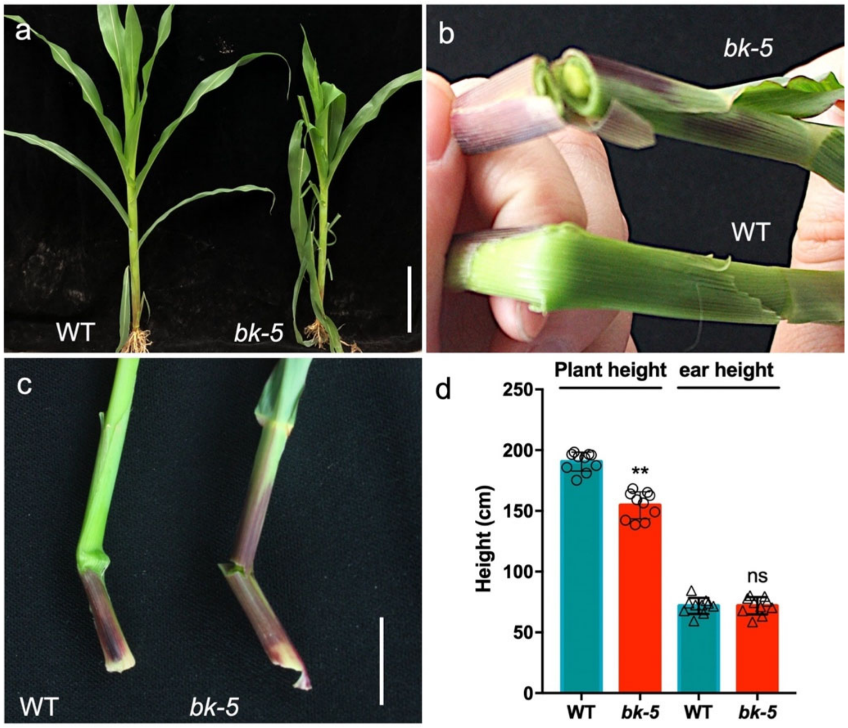


**Figure S1. The brittleness phenotype of *bk-5* in early seedling stage.** (**a**) *bk-5* and WT plants of V7 stage. Scale bar: 10 cm. (**b**,**c**) *bk-5* and WT (wild-type: RP125) plants of V3 stage. Scale bar: 2 cm. (**d**) Comparison of plant height and ear height of wild-type WT and *bk-5*. The data was presented as values are given as means ± SD and statistically calculated by Student's t test. ns, no significant change, and **(P < 0.01) indicate significant differences between RP125 and *bk-5*. The means of plant height and ear height were calculated based on ten plants.

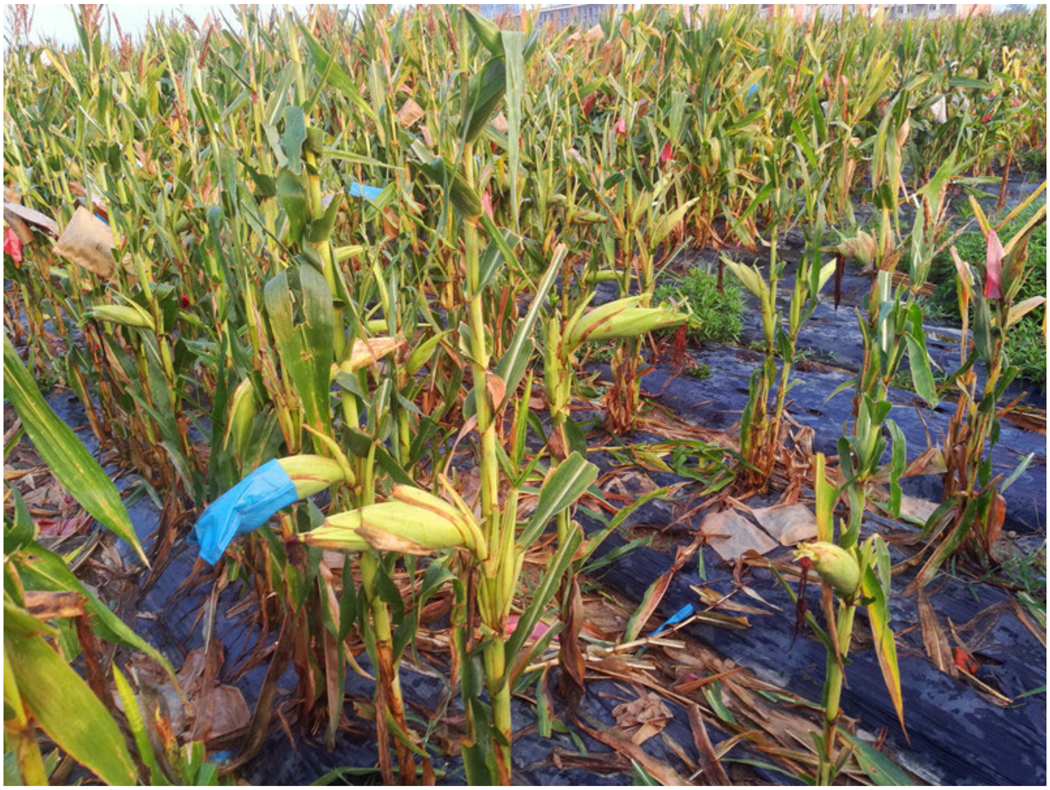

**Figure S2. The *bk-5* mutant in nature field were destroyed by the strong wind.** It was recorded in 2019, Taian, Shandong.

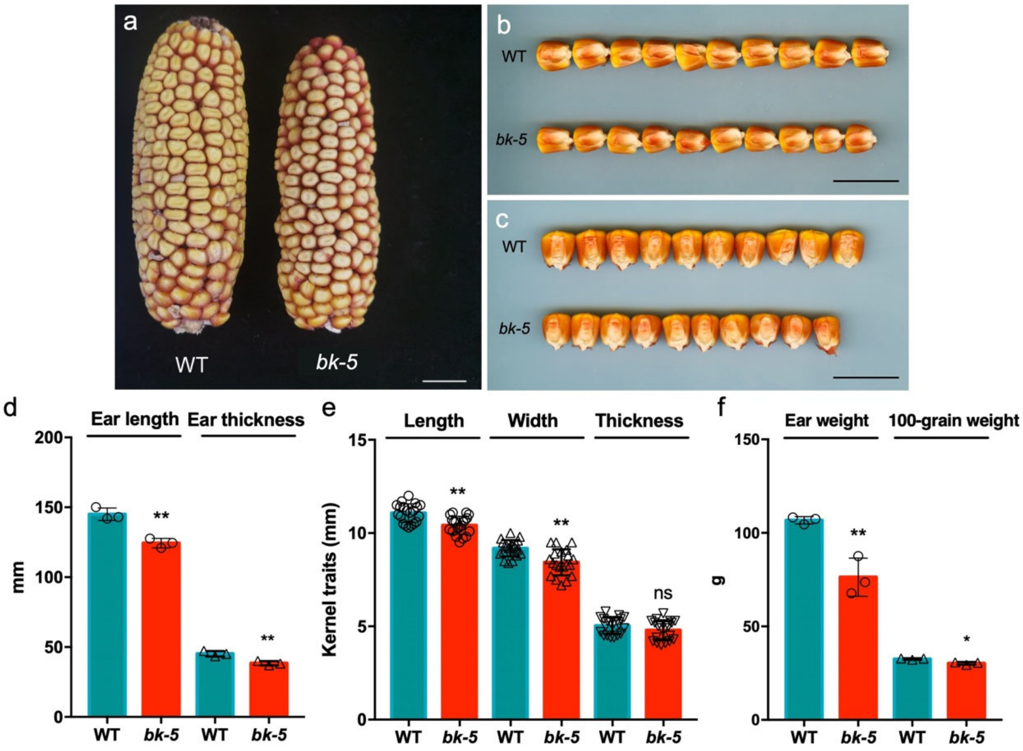


**Figure S3. Ear phenotype and agronomic traits.** (**a**) Comparison of the appearances of *bk-5* and

WT (wild-type: RP125) ears. (**b**,**c**) Phenotype comparison of kernel length (**b**) and kernel width (**c**) of WT and *bk-5*. (**d**) the agronomic traits analysis of ear length and ear thickness of WT and *bk-5*. (**e**) the agronomic traits of kernel length, kernel width, and kernel thickness of WT and *bk-5*. (**f**) The agronomic traits analysis of ear weight and 100-grain weight of WT and *bk-5*. The data was presented as values are given as means ± SD and statistically calculated by Student's t test. ns, no significant change, and *($p < 0.05$), **($p < 0.01$) indicate significant differences between RP125 and *bk-5*. The means of ear length, thickness and weight were calculated based on three ears. The means of kernel length, width and thickness were calculated based on ten kernels. 100-grain weight was calculated based on 100 kernels weight with three biological repetitions.

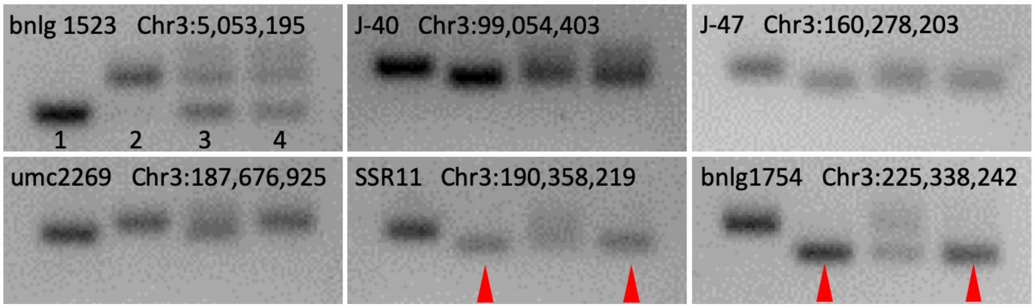


**FigureS4. Preliminary screening of linkage region using F2 segregated population mixed by BSA analysis.** The electrophoresis gel pictures of 6 selected SSR marker used for the BSA analysis were presented. For each gel, the wild type pool and mutant pool were used as the DNA template in lane 3 and lane 4; B73 and RP125 were used in lane 1 and lane 2 as the control to judge the polymorphism of molecular markers. The markers ID, and its approximate position on Chromosome were also shown on the gel. The red triangles pointed out that the mutant pool showed similar pattern as RP125 with SSR11 and bnlg1754 marker, which suggested the linkage region.

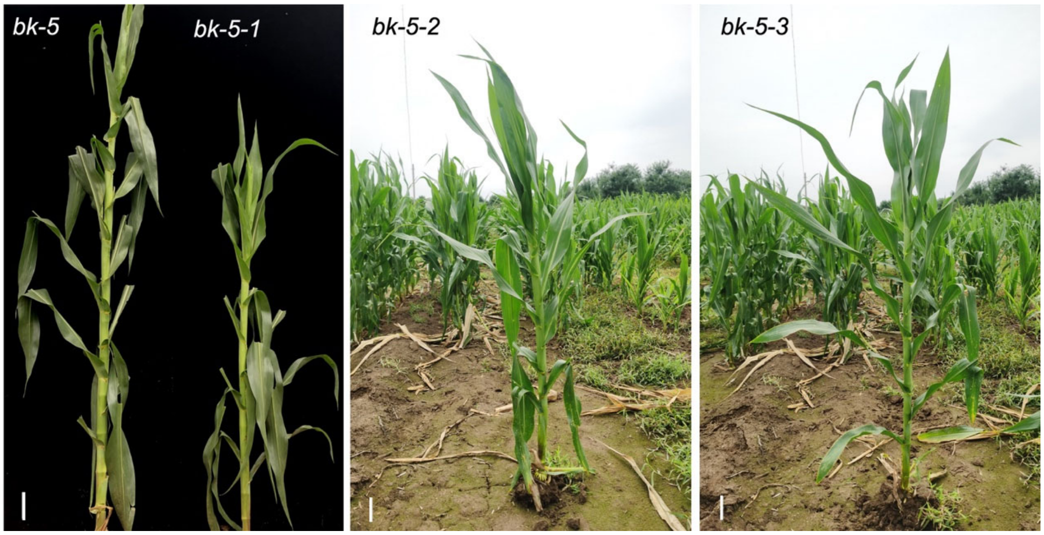


**Figure S5 The phenotype of *bk-5* and its allelic mutants *bk-5-1*, *bk-5-2* and *bk-5-3*.** Scale bar: 10 cm.

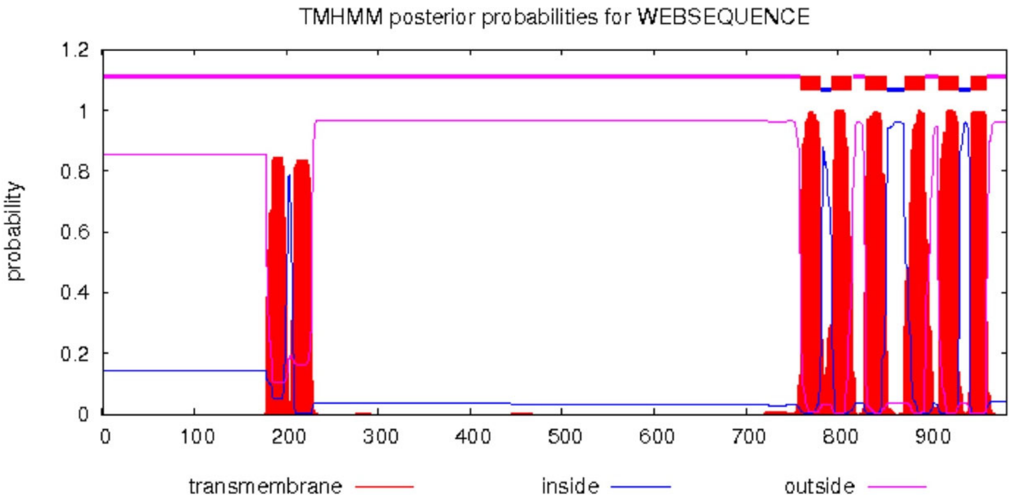


**Figure 6. Transmembrane structure analysis of BK-5 protein.** Two domains clustered near the hydroxyl terminus and six domains clustered near the carboxyl terminus. Predicted by TMHMM Server V. 2.0 (https://services.healthtech.dtu.dk/).

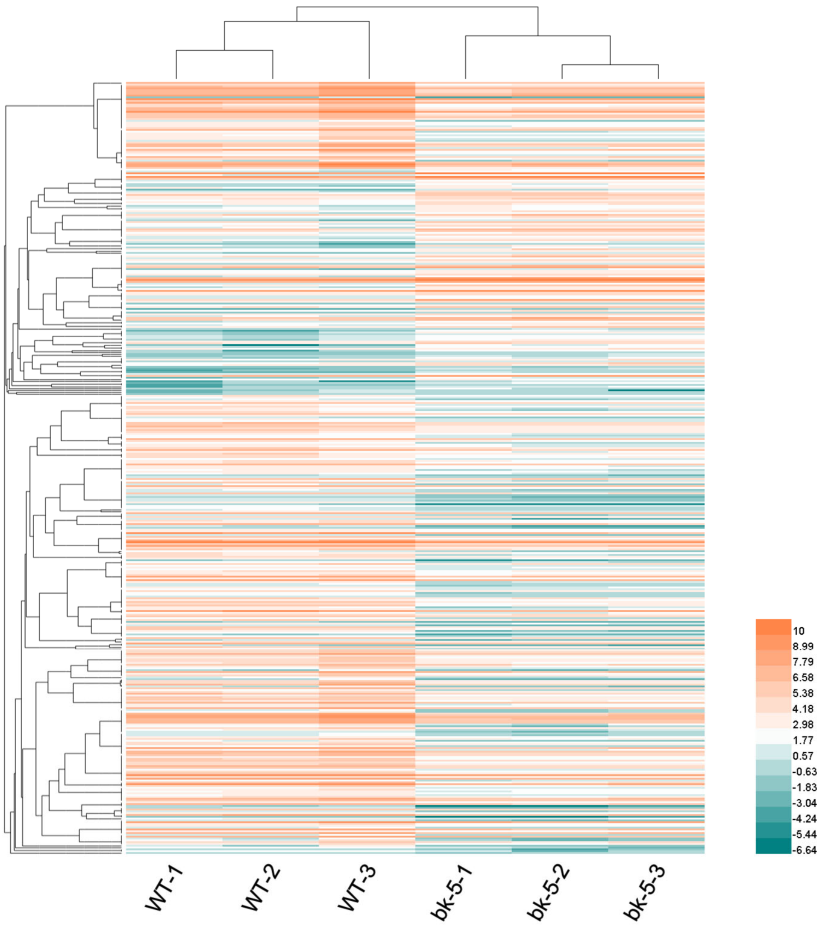


**Figure S7. The differentially expressed gene heatmap of RNA-seq data of *bk-5* and wild-type RP125**. Each row of the heat map represents the log2 fold values transformed with z score of a differentially expressed gene (green, low expression; orange, high expression). Hierarchial grouping of differentially expressed genes shows clustering. analysis by HEML. WT: wild-type.

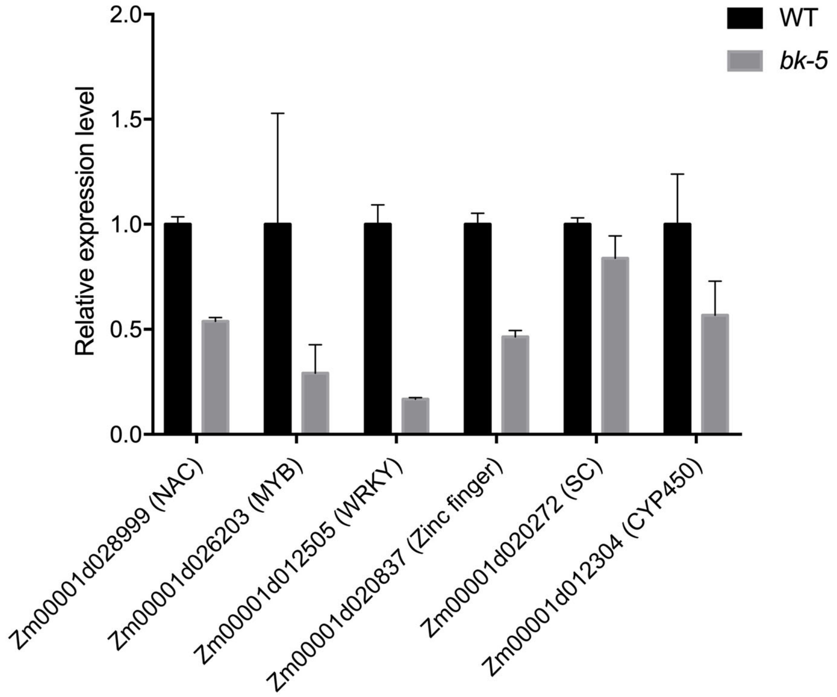


**Figure S8. One gene of each group in Fig. 7 RNA-seq data was randomly selected for quantitative verification.** The samples used were samples from the same batch of RNA-seq sequencing.18s rRNA was used as an internal control. For each RNA sample, three technical replicates of two tissue samples were performed per stage. Error bars represent standard deviation.

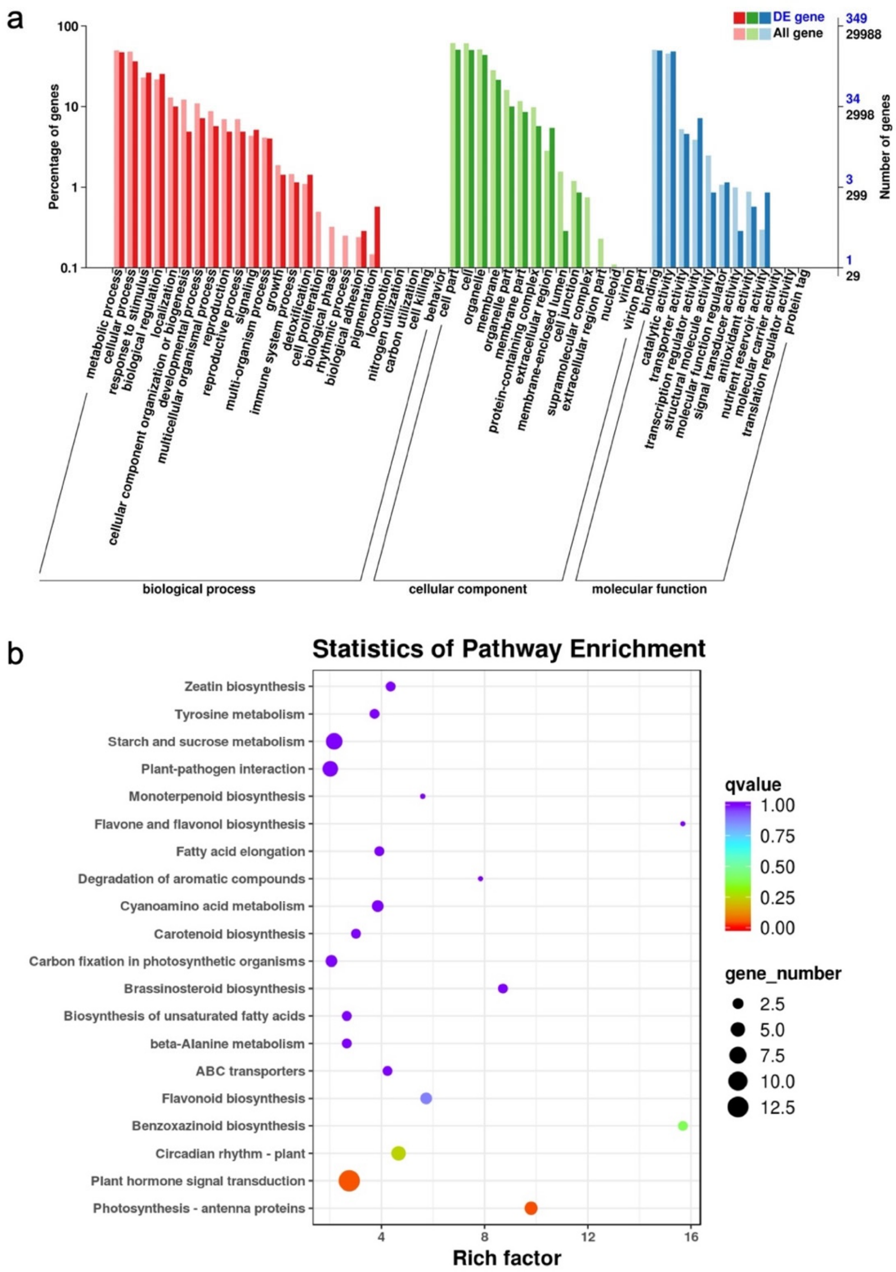


**Figure S9. Gene ontology (GO) categories and pathway enrichment of differentially expressed genes between *bk-5* and wild-type RP125.** (a) Gene ontology (GO) categories. Differentially expressed genes were annotated according to GO categories biological process, cellular component, and molecular function. Number of genes with blue font and darker color columns represent the differentially expressed genes in RP125 and *bk-5*. Number of genes with black font and light color columns represent all detected genes in RP125 and *bk-5*. (b) Pathway enrichment. the color bar means a differentially qvalue of the statistics (red, low qvalue; purple, high qvalue), and the size bar means a differentially gene number enrichment in the pathway.

**Table S1. Population segregation of mutant *bk-5*.**

| group | Normal strain | Mutants | Theoretical proportion | $\chi^2$ value ($\chi^2_{(0.05,1)}$ =3.84) |
|---|---|---|---|---|
| B73×*bk-5* | 1088 | 333 | 3:1 | 1.8581 |

**Table S2. Preliminary screening of linkage markers for mutant gene.**

| Molecular Markers | Forward primer | Reverse primer | Position (V4) |
|---|---|---|---|
| SSR11 | AATACTGGACCACCAGGCAC | CGTGGGTCACCAGGAGTC | 3: 190,358,219 |
| Indel-28 | AAAGGAGTGGGAGATGGAGC | CTGACAATGAGTCATCACATGC | 3: 192,391,935 |
| Indel-33 | GTTGTCCAGTACGTCGCCAT | ACATTCAGTCTCTGTCCACGG | 3: 206,708,232 |

**Table S3. Fine mapping molecular markers.**

| Molecular Markers | Forward primer | Reverse primer | Position (V4) |
|---|---|---|---|
| Indel-2 | TCAGATGGATAGCCGTGAGGTA | GCAGGTGAGCCACTTAAACA | 3: 199,910,233 |
| Indel-40 | GCTCCGTTTTGCCCCTCTAT | AAGCTCTGCGTCTGTCTGTC | 3: 200,440,647 |
| Indel-43 | TTTACCACACGGATGCAATGG | CGCATGCCAGTTACCAGGTG | 3: 201,379,303 |
| GG-10 | TGCCTGAAAGGTCTCAATCTCAT | TCCAGAGACCAAAGGCACTG | 3: 201,450,434 |
| SSR13 | GCTCTATGTTATTCTTCAATCGGGC | GGTCGGTCGGTACTCTGCTCTA | 3: 201,469,527 |
| Indel-41 | TTGCAACTTGTTTGGCGTGG | CCGGATGGGGATCTCGACTA | 3: 201,752,671 |
| Indel-35 | GAGAGCAAAATCCTGCCATCAG | CACACCAATCCACATGGTTCAC | 3: 202,747,558 |

**Table S4. Primer list of *BK-5* gene amplification and RT-qPCR analysis.**

| Name | Sequence |
|---|---|
| For different segment sequence of *BK-5* gene amplification: | |
| BK-5-1F | CGGAAGCTGTATCACCCCAC |
| BK-5-1R | GGAGAAGGATAGGCACCTCA |
| BK-5-2F | GCGTTTCCCAAGATCTGGTT |
| BK-5-2R | TTCAGCAATCAGTTCCAAACAA |
| BK-5-3F | TTCACTGCTACCGTGTTCCC |
| BK-5-3R | GAAGTCAGGCCCCATGAACA |
| BK-5-4F | GCGTCCCTGAATCTGCAAAC |
| BK-5-4R | GATGATGACAGCTTGGGGCT |
| For *BK-5* RT-qPCR amplification: | |
| qPCR43477-F | AAGCCTCGGCCAAAAAGACT |
| qPCR43477-R | ATATTGGGATGACCCGGGAG |
| 18S-F | CTGAGAAACGGCTACCACA |
| 18S-F | CCCAAGGTCCAACTACGAG |
